# The effect of systemic lycopene supplementation on non-surgical periodontal therapy outcomes: A clinical trial

**DOI:** 10.1038/s41405-025-00352-6

**Published:** 2025-07-16

**Authors:** Fahimeh Rashidi Maybodi, Faezeh Sadeghi Heris

**Affiliations:** Department of Periodontology, Faculty of Dentistry, Shahid Sadoughi University of Medical Sciences, Yazd, Iran

**Keywords:** Oral conditions, Periodontics

## Abstract

**Introduction:**

Some recent studies have suggested that antioxidants, particularly lycopene, may improve periodontal treatment outcomes. However, conflicting past results regarding its effects highlight the need for further research. This study aimed to evaluate the clinical effects of oral lycopene supplementation as an adjunctive therapy in nonsurgical periodontal treatment for patients with periodontitis.

**Methods:**

In this parallel clinical trial, 42 patients aged 25 to 55 with moderate to severe periodontitis and no recent history of periodontal treatment were randomly assigned to two groups after matching for mean age, sex, periodontal disease severity, and antioxidant capacity. One group received lycopene supplementation (15 mg/day) and phase I periodontal therapy. In contrast, the other group received a placebo (containing 1 g of corn starch) and phase I periodontal therapy. Periodontal parameters, including probing depth (PD), clinical attachment loss (CAL), and bleeding index (BI), as well as serum Malondialdehyde (MDA) levels, were assessed at baseline and after two months.

**Results:**

Both groups showed significant improvements in periodontal parameters after two months. However, the lycopene group demonstrated greater reductions in probing depth (*P* = 0.009), clinical attachment loss (*P* = 0.015), bleeding index (*P* = 0.237), and MDA levels (*P* = 0.199) compared to the placebo group, confirming its positive effect in reducing oxidative stress and inflammation associated with periodontitis.

**Conclusion:**

Oral lycopene supplementation was associated with better clinical outcomes than the placebo as an adjunct to nonsurgical periodontal therapy. It may be recommended as part of a periodontal treatment plan to enhance periodontitis management.

## Introduction

Periodontal diseases arise from a multifactorial interplay involving inflammatory and immunological responses that lead to dysregulation of the host’s defense against perio-pathogenic bacterial infection [1, 2]. One key contributing factor to periodontal tissue destruction is the imbalance between reactive oxygen species (ROS) and the antioxidant defense system, a condition known as oxidative stress. This imbalance results in functional and structural alterations within periodontal tissues, ultimately contributing to the progression of periodontitis [3]. In this condition, hyperactive neutrophils generate excessive reactive oxygen species, overwhelming the host’s antioxidant capacity and leading to irreversible tissue damage [4]. Over the years, various adjunctive therapies have been explored to enhance the outcomes of conventional scaling and root planning (SRP) [5, 6]. More recently, incorporating antioxidant agents has been proposed as a viable alternative to antimicrobial therapies [7]. These substances are crucial in mitigating oxidative stress, neutralizing free radicals, and potentially improving periodontal treatment outcomes by stabilizing the redox balance [8, 9].

Lycopene, a carotenoid predominantly found in red vegetables, is recognized for its powerful antioxidant properties. Among the five major carotenoids present in human plasma—alpha-carotene, beta-carotene, beta-cryptoxanthin, lutein, and lycopene—lycopene exhibits the highest antioxidant potential, exceeding that of beta-carotene and alpha-tocopherol by two- and ten-fold, respectively. Tomatoes serve as the most abundant dietary source of lycopene [10, 11].

Epidemiological studies have established a significant positive correlation between dietary lycopene intake and reduced risk of oxidative stress-related diseases, including cancer, cardiovascular disorders, asthma, arthritis, and hepatitis [10]. Furthermore, individuals suffering from inflammatory conditions exhibit lower serum lycopene (sLyco) levels than healthy individuals. Previous research suggests that higher dietary carotenoid intake is associated with a decreased incidence of various inflammatory diseases, including cardiovascular conditions [12–14]. Oxidative stress is increasingly recognized as a contributing factor to several oral conditions, including xerostomia, burning mouth syndrome, oral malignancies, and periodontitis. Given its potent free radical scavenging ability, lycopene holds therapeutic promise for periodontal disease when administered systemically or topically [15]. Its antioxidant properties effectively neutralize oxidative damage, including DNA damage caused by hydrogen peroxide (H2O2) [16–18]. Studies have also indicated that lycopene supplementation can enhance antioxidant levels in saliva, potentially exerting a protective effect on periodontal tissues [19].

Notably, lycopene has demonstrated an excellent safety profile, with no reported toxic effects, even at high doses (3 g/kg/day). This makes it a suitable candidate for adjunctive periodontal therapy [20].

Several studies have suggested the positive effects of lycopene supplementation on improving periodontal diseases [10, 19, 21]; however, some studies have not confirmed this effect [22, 23]. Given the conflicting evidence regarding the impact of lycopene supplementation on periodontal therapy, the absence of systematic reviews with conclusive findings on this topic, and the variety of doses and methods used to administer lycopene—whether as a dietary supplement, topical agent, or systemic intervention—we decided to investigate the clinical effect of systemic lycopene supplementation as an adjunct to non-surgical periodontal therapy.

## Materials and methods

The study was conducted at the Periodontics Department of the School of Dentistry, Yazd University of Medical Sciences, between October 2023 and September 2024. Ethical approval was obtained from the Shahid Sadoughi University of Medical Sciences (Ethics ID: IR.SSU.DENTISTRY.REC.1402.027), and on 2023/9/9, the study was registered in the Iranian Registry of Clinical Trials (IRCT20230830059303N1).

### Trial design

A parallel-design randomized clinical trial was conducted in which the intervention group received Lycopene supplementation for 2 months after SRP, while the control group received a placebo for 2 months after SRP. The results were reported in accordance with the Consolidated Standards of Reporting Trials (CONSORT).

### Participants, eligibility criteria. And settings

The inclusion criteria were Age range between 25 and 55 years, diagnosis of Stage II-IV periodontitis, based on the 2017 AAP classification, clinical attachment loss (CAL) > 3 mm, with evident gingival inflammation (erythema, bleeding on probing [BOP]), systemically healthy with no underlying medical conditions affecting periodontal status, no history of periodontal treatment in the preceding six months, non-smokers, no prior use of antioxidant supplementation or immunosuppressive therapy, absence of inflammatory oral mucosal conditions, such as aphthous ulcers, lichen planus, or acute hypersensitivity reactions, no use of medications known to impact periodontal health, including calcium channel blockers and immune-suppressants, no prior or current use of antioxidant supplements, such as vitamin C or melatonin, not undergoing radiotherapy or similar treatments, no use of hormonal or anti-inflammatory medications, including ibuprofen and related drugs, not allergic to tomato or its products, not pregnant or lactating, no use of antibiotics in 3 months and no current use of antibacterial mouthwashes and free from systemic conditions that may impair wound healing.

The exclusion Criteria were non-compliance with scheduled follow-up visits, inability or failure to adhere to prescribed oral lycopene supplementation guidelines, and the occurrence of any allergic reaction to the supplement despite reviewing the history of tomato allergy in the patients’ medical records.

### Informed consent

Informed consent was obtained from all subjects, including a comprehensive explanation of the study protocol, treatment objectives, potential complications, and alternative therapeutic options.

### Interventions

Eligible patients were selected sequentially from the study sample, and demographic data, including age, sex, and inclusion criteria, were collected using a researcher-designed questionnaire.

**Fig. 1:**
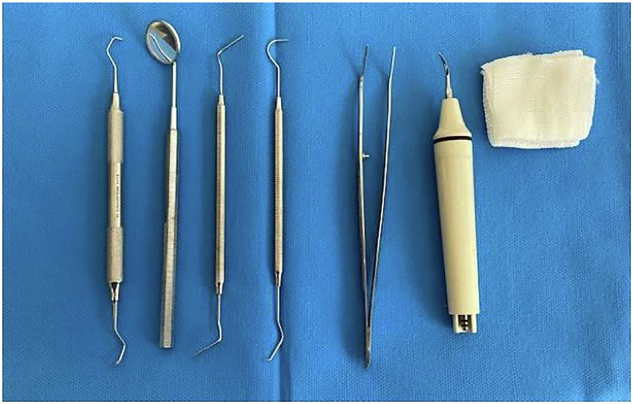
instruments by which periodontal assessment and SRP was performed.

A total of 42 eligible patients were enrolled in the study. Baseline periodontal assessments included demographic characteristics, medical and dental history, probing depth (PD), clinical attachment loss (CAL) at six sites per tooth, and bleeding index (BI). These parameters were recorded at the initial examination to confirm eligibility. All participants underwent periodontal therapy, which included standardized oral hygiene instructions emphasizing the modified Bass brushing technique (twice daily), as well as supra and sub-gingival scaling and root planning (SRP) using an ultrasonic device (Various 350, NSK, Japan). Additional scaling was performed using Gracey curettes (Hu-Friedy, Chicago, USA), followed by polishing with prophylaxis paste (Golchai, Iran). Periodontal examinations were conducted using a Williams probe and a dental mirror (Fig. 1).

As the patients were recruited from a faculty clinic that treats economically disadvantaged individuals, their routine dietary antioxidant intake was presumed to be similar. Before the intervention, both groups were matched for mean age, sex, and periodontal disease severity, as assessed by baseline CAL, PD, and BI. Socioeconomic status was also considered a matching factor to ensure comparability between the two groups regarding the dietary purchasing power of antioxidant-rich foods like fish and nuts. In addition, baseline serum levels of Malondialdehyde (MDA), a lipid peroxidation and oxidative stress biomarker, were measured and matched as closely as possible between the two groups to eliminate nutritional differences that could confound the antioxidant capacity of the individuals. The participants were randomly assigned to two equal groups of 21 patients.

**Fig. 2:**
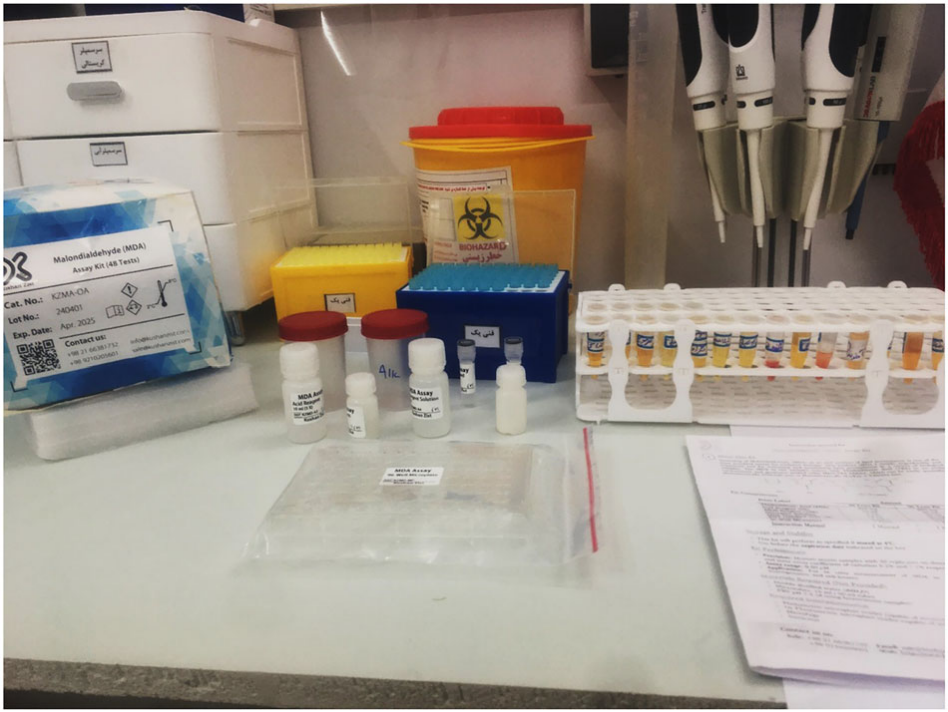
Malondialdehyde kit in left part and part of samples in right part of the pictures.

The Malondialdehyde (MDA) assay kit was used in this study. For serum sample preparation, blood is collected without an anticoagulant and left at room temperature for 30 min to clot. It is then centrifuged at 1000 × g for 15 min at 4°C. The yellow serum layer is carefully pipetted without disturbing the buffy coat layer and is kept on ice. In this assay, MDA reacts with thiobarbituric acid (TBA) under acidic conditions, forming a pink-colored MDA-TBA complex, which can be measured at 530–540 nm (photometrically) or 532/553 nm (fluorometrically). Figure 2 shows the MDA kit and part of samples (Fig. 2).

The test group received a daily dose of 15 mg Lycopene (Vitabiotics, Pharmed, Iran) for two months. Due to manufacturing constraints, placebo capsules containing corn starch were used instead of identical-looking capsules. However, because patients in the control and intervention groups did not meet, this lack of visual similarity did not affect blinding.

Patients were re-evaluated at a one-month follow-up visit to assess adherence to oral hygiene instructions and ensure compliance with the prescribed supplementation regimen. The patients were requested to continue using the supplement. After two months, all patients were recalled for a comprehensive periodontal re-evaluation, including reassessment of PD, CAL, BI, and MDA. Periodontal indices were recorded by one experienced board-certified periodontist to eliminate multi-rater bias. The post-treatment data were compared with baseline measurements, and statistical analyses were conducted to evaluate the impact of lycopene supplementation on periodontal parameters.

### Outcomes (primary and secondary)

The primary outcome of this study was to evaluate CAL change before and after the intervention. The secondary main objective of this study was to assess MDA, BI & PD probable changes according to the usage of lycopene supplementation following SRP on clinical improvement in patients with periodontitis.

### Sample size calculation

According to the results of the study by Tripathi et al. (2019) and the estimated standard deviation (SD) of SBI as 0.6, a sample size of 19 participants per group was required to achieve a minimum significant difference of 0.55 in the intervention group compared to the control group. Considering a 10% dropout rate, 21 participants per group were needed.

### Randomization

The patients were randomly assigned to the intervention and control groups by a table of random numbers generated by Random Allocation Software version 1.0 (Mahmood Saghaei, Iran).

### Blinding

The study had a double-blind design. Both Lycopene tablets and placebo capsules were in the same medication bottles. A third person not involved in the study coded the bottles as A and B and administered them to the patients. The dental clinician who measured the clinical periodontal parameters and the patients were unaware of the group allocation of bottles.

### Statistical analysis

Statistical analysis was performed using SPSS version 25 (SPSS Inc., IL, USA). The normal distribution of data was evaluated using the Kolmogorov-Smirnov test. Paired T-tests and T-tests were used to compare at the 0.05 significance level.

## Results

A total of 42 patients in 2 groups were evaluated in the study. The two groups were standardized regarding age, gender, antioxidant capacity, and baseline clinical parameters as shown in Table 1. There were 12 females (57.1%) and 9 males (42.9%) in the intervention group, and 11 females (52.4%) and 10 males (47.6%) in the control group. The two groups were not significantly different regarding gender distribution (*P* = 0.757) (Table 1).

**Table 1:** Standardization of the two groups at baseline using T-test (*n* = 21).

Group	Group	Mean	Std. Deviation	*P*-Value^*^
Age (years)	Intervention	41.43	9.796	0.116
	Control	38.62	9.413	
Baseline PD (mm)	Intervention	5.419	1.1570	0.608
	Control	5.543	1.0342	
Baseline CAL (mm)	Intervention	6.176	1.0639	0.417
	Control	6.481	1.1750	
Baseline BI	Intervention	77.48	12.813	0.404
	Control	72.81	11.474	
Baseline MDA (μmol)	Intervention	27.952	3.1244	0.744
	Control	28.395	3.0169	

*PD* Pocket depth, *CAL* Clinical attachment level, *BI* Bleeding index, *MDA* Malondialdehyde.

^*^t-test at 0.05 level of significance.

No patients were harmed during the study, and no side effects were reported.

### Subgroup analysis

The Kolmogorov-Smirnov and Shapiro-Wilk tests confirmed normal data distribution (*P* > 0.05). Table 2 presents the mean PD, CAL, BI and MDA in the two groups at baseline and 2 months after intervention. All parameters significantly decreased at 2 months compared with baseline in both groups (*P* < 0.05). The intra-group difference of the parameters is mentioned in Table 2.

**Table 2:** Mean PD, CAL, BI and MDA in the two groups at baseline and at 2 months after the intervention.

Group	Variable	Time	Mean	Standard Deviation	*P*-Value*
Intervention	PD	Baseline	5.419	1.1570	
		2 months	3.667	0.7186	
		Intra-group difference	1.7524	0.16954	0.000
	CAL	Baseline	6.176	1.0639	
		2 months	4.529	0.9317	
		Intra-group difference	1.6476	1.0337	0.000
	BI	Baseline	77.48	12.813	
		2 months	42.52	10.333	
		Intra-group difference	34.952	9.484	0.000
	MDA	Baseline	27.952	3.124	
		2 months	15.714	2.0394	
		Intra-group difference	12.2381	0.58041	0.000
Control	PD	Baseline	5.543	1.0342	
		2 months	4.452	0.9786	
		Intra-group difference	1.0905	0.17767	0.000
	CAL	Baseline	6.481	1.1750	
		2 months	5.462	1.0486	
		Intra-group difference	1.0190	0.4490	0.000
	BI	Baseline	72.81	11.474	
		2 months	41.67	11.182	
		Intra-group difference	31.143	11.019	0.014
	MDA	Baseline	28.395	3.0169	
		2 months	17.367	2.6519	
		Intra-group difference	11.0286	0.58660	0.000

^*^Paired *t*-test and *t*-test at 0.05 level of significance.

In the inter-group comparison, the improvement in PD and CAL in the intervention group was significantly greater than that in the control group (respectively *P* = 0.009 and *P* = 0.015); however, the difference in BI (*P* = 0.237) and MDA (*P* = 0.199) was not significant between the two groups.

## Discussion

This study aimed to evaluate Lycopene supplementation’s impact on periodontal treatment outcomes. The study specifically investigated the effect of systemic lycopene supplementation on the results of non-surgical periodontal therapy.

Although systematic reviews, such as Mohideen et al. (2023) [24] and López-Valverde et al. (2024) [25], have attempted to summarize lycopene’s effects on periodontal therapy, significant heterogeneity remains in study designs, dosages, and follow-up durations. The inconsistency in lycopene administration—whether dietary, topical, or systemic—prevents a definitive conclusion on its efficacy. Our study aims to address these gaps by employing a standardized protocol, ensuring uniform antioxidant intake and serum MDA assessment. This contributes to a clearer understanding of lycopene’s role in periodontal management.

A total of 21 participants were assigned to each group, a sample size derived from the 2019 study by Tripathi et al. [26] and larger than the sample sizes used in studies such as Belludi (2013) [15] and Algammal (2021) [4]. However, it was smaller than studies like Singh (2022) [19] and Ali (2021) [21]. The intervention consisted of a daily 15 mg lycopene supplement for two months, a dosage consistent with Tripathi et al. (2019) [26]. Other studies have utilized varying doses, such as Singh [19] and Belludi [15] (8–10 mg/day for two weeks) and Algammal [4] and Ambati (8–10 mg for two months) [25]. No additional antioxidant supplements were administered to isolate the effects of lycopene, unlike some previous studies that combined lycopene with vitamin C [4] or green tea extract [26]. Follow-up sessions were conducted at 4-week and 8-week intervals, consistent with studies by Tripathi [26], Algammal [4] and Ambati [25]. Some studies, such as Singh [19] and Belludi [15], employed shorter, 2-week follow-up intervals. All participants were evaluated at the 4-week mark for oral hygiene compliance and medication adherence to ensure adherence. At the 8-week endpoint, probing depth (PD), clinical attachment loss (CAL), and bleeding index (BI) were recorded, and serum MDA levels were reassessed.

In contrast to previous studies that assessed lower doses of lycopene (8–10 mg) over shorter durations (2 weeks) [15, 19], our trial implements a higher dosage (15 mg) over a two-month period to evaluate sustained effects on oxidative stress and periodontal health. Furthermore, unlike studies that combined lycopene with other antioxidants such as vitamin C [4] or green tea [26], our research examines lycopene as a standalone intervention, eliminating confounding factors and providing a clearer assessment of its independent therapeutic potential.

No adverse events or complaints were reported during the study. However, excessive lycopene consumption has been associated with skin discoloration (lycopenemia) and potential interactions with medications such as statins, antiplatelet agents, and immunosuppressants, leading to an increased risk of bleeding [27].

Periodontitis affects all age groups, with both oral and systemic health implications. While severity increases with age, early-onset aggressive forms necessitate risk recognition at all ages [28]. The WHO defines older adults as 65+ in developed and 60+ in developing countries [29]. To minimize confounding factors, our study selected participants aged 25–55, excluding younger individuals due to the aggressive nature of periodontitis in some cases, and older adults to avoid systemic comorbidities, tissue degeneration, and polypharmacy impacts. This range aligns with studies in López-Valverde’s meta-analysis [25]. Both genders were included with matched age distribution to control for oxidative stress differences, with subgroup analysis showing no significant gender-based variations. Lycopene may benefit geriatric individuals due to reduced antioxidant capacity and increased oxidative stress, but assessing its effects in older adults was beyond our study’s scope. Future research on this population is encouraged. Within 8 weeks, all clinical parameters showed a significant reduction. The reduction in PD and CAL was significantly greater in the lycopene group compared to the control. While BI also decreased more substantially in the lycopene group, the difference was insignificant. Bleeding on probing (BOP) is closely linked to inflammatory events caused by plaque accumulation. Typically, after just one week without plaque removal, the early clinical signs of inflammation**—**including gingival redness and bleeding**—**begin to appear. However, PD increase and CAL loss occur over a much longer period and do not change as rapidly. BOP is considered the best indicator of periodontal disease at the time of examination. In contrast, parameters like CAL and PD mainly reflect past destructive changes and are less influenced by the patient’s current status [30]. Given that one month had passed since the last oral hygiene evaluation (which was conducted one month after the start of the supplement), it is likely that some neglect in plaque control had occurred, leading to an increased tendency for gingival bleeding. Among studies evaluating the bleeding index, our findings are consistent with the study by Belludi (2013) [15]. However, Algammal et al. (2019) reported a significant reduction in bleeding on probing in the intervention group compared to the control group, despite the lack of close monitoring of patients’ oral hygiene during the two-month study [4]. This discrepancy may be due to the simultaneous administration of Vitamin C in Algammal’s study or better individual compliance with plaque control, even without direct supervision.

In the present study, lycopene supplementation significantly reduced serum MDA levels in the intervention group, with a greater decrease observed compared to the control group, though this difference was not statistically significant. Previous studies have shown that high salivary MDA levels are associated with periodontal inflammation, primarily due to increased reactive oxygen species (ROS) activity in this disease. Additionally, MDA levels can be lowered simply by phase one periodontal therapy (scaling and root planning), even without any adjunctive treatment, as observed in the study by Aziz et al. [31, 32].

A systematic review by Mohideen et al. (2023) demonstrated that MDA levels increase in gingival crevicular fluid (GCF), saliva, and serum in periodontitis patients compared to those with periodontal health, following a consistent pattern [24]. Our findings suggest that lycopene, through its antioxidant properties, neutralized free radicals and slightly reduced serum MDA levels as a marker of lipid peroxidation, although the standard periodontal treatment itself played a more significant role in MDA reduction. While salivary MDA measurement is convenient and non-invasive, it may be influenced by transient dietary fluctuations, hydration status, and oral hygiene practices, leading to variability in oxidative stress assessment. Serum MDA levels provide a more stable biomarker for systemic oxidative stress, reducing confounding effects and more accurately evaluating lycopene’s antioxidant potential [33]. By focusing on serum rather than salivary markers, this study enhances the reliability of its findings.

In the present study, CAL significantly decreased in both the intervention and control groups, with a significantly greater reduction in the intervention group. This finding is consistent with the results of Ambati and Algammal but contrasts with Arora’s study, where the CAL reduction between groups was not significant [4, 33, 34].

Similarly, PD showed a significant reduction in both groups, with the difference between groups being statistically significant, in line with the findings of Ambati, Algammal, and Kaur. However, in Belludi’s study, while PD decreased in both groups, the difference between groups was insignificant. This discrepancy may be attributed to the short follow-up duration (2 weeks) in Belludi’s study [4, 15, 33, 35].

### Strengths and limitations

A key strength of this research is its extended eight-week follow-up and higher dose compared with the previous studies. Additionally, baseline serum levels of MDA were measured and matched as closely as possible between the two groups to eliminate nutritional differences that could confound the antioxidant capacity of the individuals. Socioeconomic status was also considered a matching factor to ensure comparability between the two groups regarding the dietary purchasing power of antioxidant-rich foods like fish and nuts. These factors have been under-examined in past studies, despite their potential significance.

Although serum MDA provided a more stable systemic assessment than salivary biomarkers, it limited the ease of sampling.

## Conclusion

Systemic lycopene supplementation as an adjunct to non-surgical periodontal therapy led to significant clinical improvements, particularly in PD and CAL reductions. These findings suggest that lycopene may be a valuable addition to periodontal treatment protocols, but further research is warranted to validate its long-term benefits.

## Data Availability

The datasets generated and analyzed during the current study are available from the corresponding author upon reasonable request.
